# The effectiveness of an intervention in increasing community health clinician provision of preventive care: a study protocol of a non-randomised, multiple-baseline trial

**DOI:** 10.1186/1472-6963-11-354

**Published:** 2011-12-30

**Authors:** Kathleen M McElwaine, Megan Freund, Elizabeth M Campbell, Jenny Knight, Carolyn Slattery, Emma L Doherty, Patrick McElduff, Luke Wolfenden, Jennifer A Bowman, Paula M Wye, Karen E Gillham, John H Wiggers

**Affiliations:** 1 Population Health, Hunter New England Local Health District, Booth Building, Wallsend Health Services, Longworth Avenue, Wallsend, NSW, 2287, Australia; 2 Faculty of Health, The University of Newcastle, University Drive, Callaghan, NSW, 2308, Australia; 3 Faculty of Science and Information Technology, The University of Newcastle, University Drive, Callaghan, NSW, 2308, Australia; 4 Hunter Medical Research Institute, Clinical Research Centre, Level 3 John Hunter Hospital, Lookout Road, New Lambton Heights, NSW, 2305, Australia

**Keywords:** Community health, practice change, preventive care, smoking, nutrition, alcohol, physical activity

## Abstract

**Background:**

The primary behavioural risks for the most common causes of mortality and morbidity in developed countries are tobacco smoking, poor nutrition, risky alcohol use, and physical inactivity. Evidence, guidelines and policies support routine clinician delivery of care to prevent these risks within primary care settings. Despite the potential afforded by community health services for the delivery of such preventive care, the limited evidence available suggests it is provided at suboptimal levels. This study aims to assess the effectiveness of a multi-strategic practice change intervention in increasing clinician's routine provision of preventive care across a network of community health services.

**Methods/Design:**

A multiple baseline study will be conducted involving all 56 community health facilities in a single health district in New South Wales, Australia. The facilities will be allocated to one of three administratively-defined groups. A 12 month practice change intervention will be implemented in all facilities in each group to facilitate clinician risk assessment of eligible clients, and clinician provision of brief advice and referral to those identified as being 'at risk'. The intervention will be implemented in a non-random sequence across the three facility groups. Repeated, cross-sectional measurement of clinician provision of preventive care for four individual risks (smoking, poor nutrition, risky alcohol use, and physical inactivity) will occur continuously for all three facility groups for 54 months via telephone interviews. The interviews will be conducted with randomly selected clients who have visited a community health facility in the last two weeks. Data collection will commence 12 months prior to the implementation of the intervention in the first group, and continue for six months following the completion of the intervention in the last group. As a secondary source of data, telephone interviews will be undertaken prior to and following the intervention with randomly selected samples of clinicians from each facility group to assess the reported provision of preventive care, and the acceptability of the practice change intervention and implementation.

**Discussion:**

The study will provide novel evidence regarding the ability to increase clinician's routine provision of preventive care across a network of community health facilities.

**Trial registration:**

Australian Clinical Trials Registry ACTRN12611001284954

**Universal Trial Number (UTN):**

U1111-1126-3465

## Background

The primary behavioural risks for the most common causes of mortality and morbidity in developed countries are tobacco smoking, poor nutrition, risky alcohol use, and physical inactivity [1-3]. In Australia, 20% of adults are current smokers, 13% consume alcohol at risky levels, 86% have inadequate vegetable consumption, 46% have inadequate fruit consumption, 35% are sedentary, and a further 37% have low levels of physical activity [4]. Furthermore almost all adults (92%) have at least one chronic disease risk, and 44% have three or more such risks [5-7].

Cochrane review evidence [8-12] supports the efficacy of clinician delivery of care to increase: smoking cessation [11,12], the consumption of fruit and vegetables [8], to reduce at-risk alcohol consumption [9], and to increase physical activity [10]. Consistent with such evidence, the routine delivery by health care providers of preventive care incorporating risk assessment, brief advice and referral for such behavioural risks has been recommended in a number of countries [13-23].

In a range of countries including Australia, community health services represent a key primary health care setting for the provision of preventive health care [19,20,24-30]. Community health services are the second largest provider of health care to the Australian population, providing approximately 8.6 million occasions of service annually in one state alone [18,29,31]. In Australia, community health services provide a diverse range of care types, including: community nursing, allied health, community child and family health, diabetes services, aged care, post acute care, mental health, drug and alcohol, and sexual assault care; and are delivered by a variety of providers, most commonly nurses and allied health professionals [29].

There is limited evidence regarding the provision of preventive care for smoking, poor nutrition, alcohol misuse and physical inactivity risks in community health services in Australia and elsewhere [27,32-34]. In a study of three Australian community health teams, Laws et al (2009) found the mean proportion of clients interested in changing their behaviour who reported receiving brief advice or referral regarding these risks ranged between 43-66% [27]. Another Australian study [35] reported low levels of brief advice ranging between 2% and 3% for these four risks. One international study has also reported low levels of assessment (34%), brief advice (46%) and follow-up (0%) for smoking [34]; and another found similarly low levels for smoking assessment (12%) and brief advice (10%), and physical activity brief advice (15%) [33]. Such data suggest that the delivery of preventive care is less than optimal and that the intended clinical and population health benefits of such care are currently unrealised.

The authors located few controlled studies designed to increase the provision of any form of preventive care in community health settings [34-36]. The identified studies were undertaken across one home health care agency [34], 12 Aboriginal community health centres [35], and 42 pre-natal care clinics [36]. The interventions involved one [34], three [36], or six [35] practice change strategies, and reported significant improvements for at least one outcome based either on a medical record audit [35], or client [36] or clinician self-report [34,36]. One study reported the provision of brief advice only [35], while the other two both reported on assessment, brief advice and follow-up. None of these studies reported referral to another service. Two of the studies reported a significant increase in the prevalence of care at follow-up compared to baseline [34] or a control group [35], with a 41% increase for risk assessment [34], increases ranging from 3% to 40% for brief advice [34,35], and an increase of 17% for follow-up [34]. The remaining study reported significant increases in mean scores for assessment, brief advice and follow-up [36].

Practice change theories [37-40] and evidence from reviews of practice change interventions in the broader primary care setting suggests that a multi-strategic approach is most likely to increase clinician care provision [18,41-46]. Intervention strategies that have been shown to be effective in changing clinical practice [22,32], are those that address: local consensus processes and organisational leadership; access to enabling organisational systems (including systems for the process, structure and content of care); educational meetings and ongoing support for clinicians; audit and feedback; and distribution of educational materials and patient resources [42-45]. The implementation of such strategies has also been shown to be effective in enhancing the provision of preventive care regarding smoking [18,47,48], and inadequate diet and physical activity [21], by general practitioners and hospital-based clinicians.

## Methods/Design

### Study aim

The primary aim of the study is to assess the effectiveness of a multi-strategic practice change intervention in increasing clinician provision of recommended preventive care (risk assessment, brief advice, and referral) [21-23,49] for each of four chronic disease health risk behaviours (smoking, inadequate fruit and vegetable consumption, risky alcohol use, and inadequate physical activity) across a network of community health facilities.

### Study design and setting

A multiple baseline study [50] will be conducted involving all 56 community health facilities in a single health service in New South Wales, Australia. The service provides comprehensive healthcare to approximately 840000 people residing in metropolitan, regional, rural and remote location, and employs over 1400 community health clinicians who see in total approximately 57000 clients per year.

**Figure 1:**
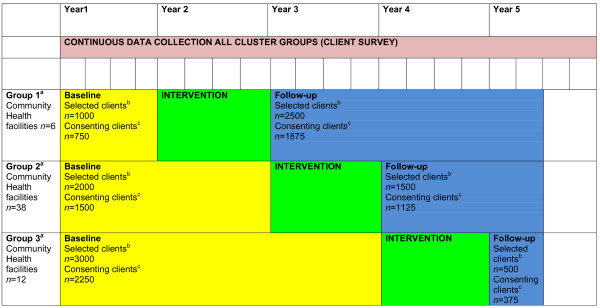
**Overview of multiple baseline study showing three phase rollout, intervention periods and outcome measurement**. ^a ^Not randomly allocated. ^b ^Calculated on 50 weeks of data collection per year. ^c ^Based on a 75% consent rate to client survey.

The study will involve the sequential implementation of a 12 month practice change intervention in three administratively defined groups of community health facilities (Figure 1). The intervention will be implemented in a non-random sequence across the three facility groups.

The multiple baseline study design provides a number of advantages that are relevant to the conduct of complex health service research interventions [51,52]. Firstly, the design provides the opportunity for all facilities to participate in the intervention and all clients to receive the intervention (a key motivational requirement for clinician engagement) [52]. Secondly, its sequential implementation allows for any effect of extraneous variables on the outcomes to be monitored [52]. Finally, the design addresses the practical difficulty of recruiting a sufficient number of similar community health facilities, and is more efficient because each group is used as its own control [51,52].

The study is funded by the Hunter Medical Research Institute, and a partnership grant from the National Health and Medical Research Council (application ID: APP1016650) in partnership with the Hunter New England Local Health District. Ethical approval to conduct the study has been obtained from the Hunter New England Human Research Ethics Committee (approval No. 09/06/17/4.03) and University of Newcastle Human Research Ethics Committee (approval No. H-2010-1116).

### Sample participants and recruitment

#### Community health facilities

The facilities offer a common range of community based prevention, early intervention, assessment, treatment, health maintenance and continuing care delivered by a variety of providers [24]. The forms of the health services that can be provided by such facilities include: community nursing, allied health, community child and family health, mental health, drug and alcohol, diabetes services, and aged care. The following specific forms of services will be excluded: inpatient services, sexual assault, palliative care, genetics services, and child protection services (parents).

#### Clients

All adult clients who have at least one visit to a community health facility for the above types of services within the prior two weeks, and who meet the following inclusion criteria will be eligible to participate in the data collection: over 18 years of age; speak English; mentally and physically capable of completing an interview; not determined by clinician discretion as inappropriate to contact; not involved in another community health care focused survey; and not living in aged care facilities or gaol.

Each week 20 eligible clients who have not previously participated in the study will be randomly selected from the electronic medical records of each of the three groups of facilities (60 clients per week in total). Selected clients will be mailed an information letter then contacted by phone and asked to participate in a telephone interview. As indicated in Figure 1, the three groups will have different numbers of clients participating in the surveys in the baseline and follow-up periods due to the staggered multiple baseline design. The study is anticipated to recruit 750, 1500 and 2250 clients in the baseline period for groups 1, 2 and 3 respectively, and 1875, 1125 and 375 clients in the follow-up period for these groups.

#### Clinicians

All clinical staff responsible for providing the eligible forms of care within the community health facilities and who: have at least 10 appointments with adult clients (> 18 years) within the last two months; have been employed for at least three months; and are not contractors will be eligible to participate in a telephone survey.

During the baseline period for group 1 and immediately following the intervention for group 3, a random sample of eligible community health clinicians from each group will be selected from health service records to participate in a survey (a minimum of approximately 100 clinicians per group). Clinicians will be mailed an information letter, and called during work hours within the following four weeks to participate in the survey.

#### Model of preventive care

Recommendations regarding clinician provision of preventive care commonly suggest that such care involves the provision of brief advice or care according to the '5 A's' behavioural counselling framework [14-18]. However, it has been recommended that the 5A's model be shortened to 2 As and an R (ask, advise and refer) due to competing clinical priorities and the brevity of the clinician-client contact [21-23,49]. Such an approach enables all the elements of the 5A's to be provided, but not necessarily within the original service, and thus addresses the capacity limitations of clinicians [18]. Such an emphasis on referral enhances client access to specialist preventive care referral services such as telephone helplines [21-23,53,54]. Based on this recommendation, and national guidelines that recommend clinician provision of preventive care [15,16,19,20], the current study will support the provision of the following elements of care: assessment of all risks, and for all relevant risks, the provision of brief advice and offering a referral to general practitioners/Aboriginal Medical Service providers, telephone helplines or other care providers.

#### Assessment

As recommended in preventive care guidelines [15,17,21,23] clinicians will assess a client's risk status for the following: smoking of any tobacco products [55], consuming more than two drinks on a regular drinking day or four or more drinks on any one occasion [56], undertaking less than 30 minutes of physical activity on at least five days of the week [57], or consuming less than two serves of fruit or five serves of vegetables per day [58].

#### Brief advice

As recommended in preventive care guidelines and policies [15,16,19,20], clinicians will provide clients with brief advice suggesting that they modify their identified risks.

#### Referral

Clinicians will offer a referral for 'at risk' clients to specialist risk reduction services where they exist, (e.g. NSW Quitline for smokers, NSW Get Healthy Information and Coaching Service for clients with inadequate fruit or vegetable consumption, or physical inactivity) or a general practitioner/Aboriginal Medical Service provider (for clients at risk for alcohol misuse) [53,54].

#### Clinical practice change intervention

Multiple practice change strategies, based on practice change theory [37-40] and research demonstrating effectiveness of strategies in modifying clinical practice [46-48,59-65],will be delivered to the community health facilities by the Health District Population Health service to support clinicians to provide assessment, brief advice and referral (for further definitions of terms see Additional file 1) [66].

#### Local opinion leaders and consensus processes

Oversight and corporate support of the intervention will be via a purpose specific Taskforce involving health service executives. To increase intervention adherence, consensus for the model of preventive care will be sought and formalised through the development of a Preventive Care Policy, applicable to all clinicians. An Aboriginal Advisory group will be established to provide oversight regarding the cultural appropriateness of the intervention. At the facility level, consultation with managers and clinicians will be undertaken.

#### Enabling clinical and management organisational systems

The electronic medical record system, utilised across all sites within the area health service to record care provided by clinicians, will be modified to standardise: client eligibility for risk assessment, brief advice and referral; prompt clinician delivery of preventive care; provide assessments and suggestions for provision of brief advice based on age; record the provision of each form of care; enable the production of an automated tailored letter for clients' general practitioner and a similar letter for the client; and enable the generation of preventive care delivery performance reports for health service managers. A hard copy preventive care checklist will be provided to facilitate care provision and recording for use in home visits.

#### Clinician and manager educational meetings; educational outreach visits and academic detailing; and client and clinician practice change resources

##### Clinician and manager educational meetings

The Health District Population Health service will provide current clinicians with competency based online training. The modular training will take approximately two hours in total, involve didactic and quiz based components, and will address the model of preventive care, the standards of delivery for each aspect of preventive care, and how to record care delivery in the electronic medical record. New clinicians will attend such training as a component of new staff orientation procedures.

All managers will be required to complete two hours of face to face training regarding leadership of the initiative and the conduct of performance audit and feedback.

##### Educational outreach visits and academic detailing

Practice change support officers will be allocated to each community health facility to facilitate implementation of practice change strategies and clinician provision of preventive care. The support officers will provide a minimum of one face to face visit per month during the intervention period and fortnightly telephone support for managers to support the implementation and maintenance of the intervention.

##### Clinician practice change resources

An email helpline, an internet site that includes all clinician support resources (e.g. referral forms, hardcopy assessment tool for home visits, electronic medical records data entry guide), a clinician resource pack (also containing these support resources), and referral resources will be provided. Managers and clinicians will be provided with newsletters, tips and updates sheets, and a workstation reminder to prompt and provide additional information and solutions to problems.

##### Audit and feedback

Monthly performance reports describing the prevalence of clinician preventive care provision will be provided to managers via email. The reports will provide information regarding care delivery at the individual service, facility, and larger administrative unit levels. Feedback and advice regarding care delivery performance will be provided by the support officers.

##### Community promotion

General practice organisations will be regularly briefed regarding the initiative, and articles describing the intervention will be published in their newsletters.

To increase awareness of the initiative among clients and community members, general community and Aboriginal-focused media releases will be issued at the commencement of the initiative in each facility. A series of posters and an Aboriginal community brochure will be disseminated by health facilities to further promote awareness of the initiative.

### Comparison sites

Prior to the implementation of the intervention in each group of facilities, usual preventive care practice change strategies will be utilised in the comparison sites. Some intervention strategies will be applied across the entire district (all groups) but not enacted until each groups' intervention period commences (e.g. the decision on the model of preventive care, and the changes to the electronic records and policy). While some strategies will only be implemented during the intervention period (such as educational outreach visits and academic detailing; and community promotion), some clinical practice change elements will remain after the research project concludes to increase the sustainability of clinician provision of preventive care (e.g. leadership and consensus processes, organisational systems change, and audit and feedback).

### Contamination

The risk of intervention contamination between groups is considered to be low given that implementation of the practice change strategies will occur in a controlled sequential fashion across the administratively separate community health facility groups. Changes in the delivery of preventive care prior to the implementation of the intervention in each group will be used to examine the extent of contamination.

### Data collection procedures

The primary source of data regarding the delivery of preventive care will be collected from client computer-assisted telephone surveys. A secondary source of data for the primary outcome will also be collected from clinician computer-assisted telephone surveys. Data regarding acceptability of preventive care delivery will be collected from the client and clinician surveys. The surveys will be pilot tested and administered by trained interviewers.

Clients will be blind to the fact that they are part of an intervention or comparison group of a research trial. Clinicians will be aware of their allocation to either the control or intervention periods.

Additional data regarding client, clinician and facility characteristics will be obtained from medical and health service records.

Measurement of the implementation of the clinical practice change strategies will occur based on information recorded in project management records.

### Measures

#### Client characteristics

Throughout the study period, consenting clients will be asked in the telephone interview to report their: current employment status (employed, not working, retired, other); Aboriginal or Torres Strait Islander Origin status (yes, no); marital status (not living with a partner, living with a partner); highest level of education achieved (some high school or less; completed high school; technical certificate or diploma; University, or college degree or higher); and whether in the last two months the client had any conditions for which they needed to take medication or receive medical attention (yes, no/don't know) [47].

The following client information will be obtained from the electronic medical records at the time of their random selection: age, gender; country of birth; postcode; and number of visits to that service in the prior 12 months.

#### Client risk status

Clients will be asked to report their risk status for each of four risks in the month before seeing the service: whether they were a smoker of any tobacco products (daily; at least once a week; less than once a week; quit less than four months ago; quit four months or more ago; never smoked); the number of serves of fruit (0, 1, 2 or more, don't know) and vegetables (0, 1, 2, 3, 4, 5 or more, don't know) usually eaten per day; how often they had a drink containing alcohol (never, monthly or less, 2-4 times a month, 2-3 times a week, 4 or more times a week, don't know), the number of standard drinks they had on a typical drinking day, (1 or 2, 3 or 4, 5 or 6, 7 to 9, 10 or more, don't know), and how often they had four or more standard drinks on any one occasion (never, less than monthly, weekly, daily or almost daily, don't know); and how many days a week they usually did 30 minutes or more of physical activity (0, 1, 2, 3, 4, 5 or more, don't know, unable to).

The survey items for each risk are based on validated items from recommended assessment tools [67-70], and have been used in previous surveys [27,71]. Based on national guidelines [55-58], risk will be defined as: smoking any tobacco products [55], eating less than two serves of fruit or five serves of vegetables per day [58], drinking more than two standard drinks a day or four or more standard drinks on any one occasion [56], and engaging in less than 30 minutes of physical activity on at least five days of the week [57].

#### Preventive care receipt

##### Assessment

Clients will be asked whether, during an appointment with the service a clinician asked: if they smoke any tobacco products; how many vegetables and fruit they eat; how much alcohol they drink; and how much physical activity they do (yes, no, don't know).

##### Brief advice

Clients identified as being 'at risk' for any risk will be asked whether a clinician advised them to: quit smoking, or advised about Nicotine Replacement Therapy [72]; eat more fruit, or vegetables [58]; reduce how much alcohol they consume [69], and do more physical activity [73] (yes, no, don't know).

##### Referral

'At risk' clients will be asked whether a clinician asked if their general practitioner/Aboriginal Medical Service provider could be informed of the consultation; offered to arrange a referral to the Quitline for smoking, or the Get Healthy Information and Coaching service for inadequate fruit and vegetable consumption and/or physical inactivity; or advised them to visit their general practitioner/Aboriginal Medical Service provider (yes, no, don't know) for risky alcohol use.

##### Acceptability of preventive care delivery

Clients will be asked if clinician provision of risk assessment, and for 'at risk' clients, brief advice and referral, was acceptable for each risk individually and for all risks simultaneously (strongly disagree, disagree, unsure, agree, strongly agree).

##### Clinician characteristics and risk status

Consenting clinicians will be asked to report: their age (< 40, 40-49, 50-59, 60+); Aboriginal or Torres Strait Islander Origin status (yes, no); current employment status (full time, part time, casual, other); number of years in their discipline (< 1, 1-2, 3-4, 5-9, 10+), and years working in community health (< 1, 1-2, 3-4, 5-9, 10+). Clinicians will be asked to report their risk status for each of the four risks in the past month, using the same questions and response categories as previously described for clients.

The following clinician information will be obtained from health service records: position, team, professional type, postcode of service, and gender.

#### Preventive care delivery

##### Assessment

Clinicians will be asked to estimate the proportion of their new adult clients from the previous two months for whom they had assessed smoking status, fruit and vegetable intake, alcohol consumption, and current physical activity status (for each risk: 0 to 100%, don't know).

##### Brief advice

In the telephone interview, clinicians will be asked to report on the proportion of 'at risk' clients that they advised: to quit smoking, to eat more fruit and/or vegetables, to reduce alcohol intake, and to increase physical activity levels (for each risk: 0 to 100%, don't know).

##### Referral

Clinicians will be asked to report the proportion of clients for whom they informed the client's general practitioner/Aboriginal Medical Service provider of the client's risk status and care received (0 to 100%, don't know). In addition, clinicians will be asked to report the proportion of clients at risk for smoking, inadequate fruit or vegetable consumption or physical inactivity with whom they spoke to about the recommended telephone service, or their general practitioner/Aboriginal Medical Service provider for risky alcohol use (for each risk: 0 to 100%, don't know).

##### Acceptability of preventive care intervention

To determine acceptability of the intervention, the clinician telephone survey will assess: clinicians perceived barriers to the provision of preventive care for the four individual risks, and the four risks simultaneously; the appropriateness, acceptability and utility of the preventive care elements; and the usefulness of the clinical practice change strategies implemented (strongly agree, agree, unsure, disagree, strongly disagree).

#### Practice change intervention delivery

Data regarding the following measures of clinical practice change intervention delivery will be obtained, including: meetings of advisory groups, number of facility contacts; number of clinicians completing training, provision of performance reports.

#### Community health service characteristics

Data regarding community health facility descriptors for each client will be obtained from health service records regarding: service team name (e.g. social work), group (1, 2, 3) and service type (community nursing, allied health, community child and family health, diabetes services, aged care, and other services).

### Sample size

Assuming a baseline prevalence of 50% for all forms of preventive care delivery (worst case scenario), the study will have 80% power to detect a difference of between 4.7% to 21.8% in the assessment of each health risk between baseline and follow-up for the three groups at the 1% significance level. Power estimates for the provision of brief advice and referral for all four risk behaviours are conservatively based on the expected number of smokers; as smoking is the least prevalent risk behaviour [1]. The sample in the third group of facilities (which has the shortest follow-up period) is expected to include approximately 450 (20%) smokers in the baseline period and 75 smokers during the follow-up period. With a brief advice prevalence of 50% at baseline, the study will have over 80% power to detect a 20.9% improvement in the prevalence of such care (i.e. a change from 50.0% to 70.9%). Similarly, with an estimated prevalence of 50% for offering a referral prior to the intervention, the study will have over 80% power to detect a 20.9% improvement in prevalence of such care (i.e. a change from 50.0% to 70.9%).

### Statistical analysis

#### Sample characteristics

Clients and clinicians completing the surveys will be compared to eligible non-participants in terms of characteristics and clinical descriptors using chi-square analyses. The characteristics of clients and clinicians obtained from their respective surveys at baseline will be compared with equivalent data at follow-up.

#### Preventive care delivery

##### Client data

For each outcome, evaluation of intervention effectiveness will involve fitting a segmented logistic regression model, including separate intercepts and slopes at baseline, during the intervention and at follow-up (the three "segments") [74,75]. A statistically significant coefficient for the intercept and/or slope during the intervention or follow-up will indicate a change in the outcome measure following the intervention for each group. Standard errors of coefficients, and hence p-values and confidence intervals, will be estimated using bootstrapping [76]. In the first set of models the outcome of interest will assess client reported assessment of risk behaviour. The second set of models will be restricted to those subjects who report a risk behaviour, with separate models assessing whether or not the community health clinician provided brief advice, or offered a referral.

##### Clinician data

Baseline and follow-up clinician data will be analysed using logistic regression analyses to detect patterns of clinician provision of preventive care at baseline and following the intervention.

##### Acceptability

Client and clinician reported acceptability data will be reported using simple descriptive statistics.

## Discussion

This study aims to assess the effectiveness of a multi-strategic intervention in increasing community health clinician provision of preventive care for each of the four most common chronic disease risk behaviours. The study will enhance the currently limited experimental evidence regarding the effectiveness and acceptability of a multi-strategic practice change intervention in encouraging primary health care providers to address multiple chronic disease risks simultaneously, and in enhancing the provision of preventative care across a large network of community health facilities.

## Competing interests

The authors declare that they have no competing interests.

## Authors' contributions

First author KM led the development of this manuscript. Authors JK, MF, KG and JW conceived the intervention concept. Authors JW, PM, EC, LW, JB, MF, PW, KG, and JK secured grant funding from the National Health and Medical Research Council. All authors contributed to the research design and trial methodology and contributed to, read and approved the final version of this manuscript.

## Pre-publication history

The pre-publication history for this paper can be accessed here:

http://www.biomedcentral.com/1472-6963/11/354/prepub

## Supplementary Material

Additional file 1 **Table 1. Intervention strategies to change health professional practice^a^**. This file contains a table of definitions of intervention strategies to change health professional practice, modified from the EPOC taxonomy of professional quality improvement strategies. Click here for file
