# Supplementary material for: The effectiveness of an intervention in increasing community health clinician provision of preventive care: a study protocol of a non-randomised, multiple-baseline trial
Source: BMC Health Serv Res. 2011 Dec 30;11:354. doi: 10.1186/1472-6963-11-354 (PMC3268753; doi:10.1186/1472-6963-11-354)
Supplement: Additional file 1 — Table 1. Intervention strategies to change health professional practicea. This file contains a table of definitions of intervention strategies to change health professional practice, modified from the EPOC taxonomy of professional quality improvement strategies. [file 1472-6963-11-354-S1.PDF]

Table 1. Intervention strategies to change health professional practice[a]

Interventions	Definition
Distribution of educational materials	Published or printed recommendations for clinical care including clinical practice change guidelines, delivered personally or through mass mailings.
Educational meetings	Healthcare providers who have participated in conferences, lectures, workshops, or traineeships.
Local consensus processes	Inclusion of participating providers in discussion to ensure that they agreed that the chosen clinical problem was important and the approach to managing the problem was appropriate.
Educational outreach visits and academic detailing	Use of a trained person who met with providers in their practice settings to give information with the intent of changing the provider's practice. The information given may have included feedback on the performance of the provider(s).
Local opinion leaders	Use of providers nominated by their colleagues as "educationally influential."
Patient mediated interventions	New clinical information (not previously available) collected directly from patients and given to the provider.
Audit and feedback	Any summary of clinical performance of health care over a specified period of time. The summary may also have included recommendations for clinical action. The information may have been obtained from medical records, computerised databases, or observations from patients.
Reminders	Patient or encounter specific information, provided verbally or on paper, or on a computer screen, which is designed or intended to prompt a health professional to recall information, including computer aided decision support.
Marketing	A survey of targeted providers to identify barriers to change and subsequent design of an intervention that addresses identified barriers.
Professional	Individual behaviour (distributing educational materials) and organisational interventions (local consensus processes).
Financial	Includes individual and organisational incentives and environmental restructuring (changing the available products).
Organisational	Includes input (changing skill mix), processes (communication) and effects (satisfaction of providers). Influencing the organisation of services, including the process of care (delegation of tasks), the structure of care (the follow-up system) and the content of care (health charts, flow sheets).
Regulatory	Includes legal (changes in patient liability) and social influence (peer review).
Patient resources[b]	Distribution or addition of resources that may aid discussions of risk factors, or allow previously unavailable options for preventive care, including flipcharts, educational resources for patients and referral opportunities (e.g. quitlines).
Ongoing support[b]	Email, telephone or face to face communications which provided support and advice, responded to questions, or problems.

[a]Modified EPOC taxonomy of professional quality improvement strategies[66]
[b]Intervention strategies not covered by EPOC criteria.
